# Genetic factors affecting dopaminergic deterioration during the premotor stage of Parkinson disease

**DOI:** 10.1038/s41531-021-00250-2

**Published:** 2021-11-26

**Authors:** Myung Jun Lee, Kyoungjune Pak, Han-Kyeol Kim, Kelly N. Nudelman, Jong Hun Kim, Yun Hak Kim, Junho Kang, Min Seok Baek, Chul Hyoung Lyoo

**Affiliations:** 1 Department of Neurology, Pusan National University Hospital, Pusan National University School of Medicine and Biomedical Research Institute, Busan, Republic of Korea; 2 Department of Nuclear Medicine, Pusan National University Hospital, Pusan National University School of Medicine and Biomedical Research Institute, Busan, Republic of Korea; 3 Department of Neurology, Gangnam Severance Hospital, Yonsei University College of Medicine, Seoul, Republic of Korea; 4 Department of Medical and Molecular Genetics, Indiana University School of Medicine, Indianapolis, IN USA; 5 Department of Neurology, National Health Insurance Service Ilsan Hospital, Goyang, Gyeonggi-do Republic of Korea; 6 Department of Anatomy, Pusan National University School of Medicine and Biomedical Research Institute, Yangsan, Gyeongsangnam-do Republic of Korea; 7 Department of Biomedical Informatics, Pusan National University School of Medicine, Yangsan, Gyeongsangnam-do Republic of Korea; 8 Interdisciplinary Program of Genomic Data Science, Pusan National University, Busan, Republic of Korea; 9 Department of Neurology, Wonju Severance Christian Hospital, Yonsei University Wonju College of Medicine, Wonju, Gangwon-do Republic of Korea

**Keywords:** Parkinson's disease, Neurodegeneration

## Abstract

To estimate dopaminergic dysfunction in patients with Parkinson disease (PD) during the premotor stage and to investigate the effect of genetic factors on the trajectories. Using longitudinal dopamine transporter single-photon emission computed tomography data from 367 sporadic PD (sPD), 72 *LRRK2* (G2019S), and 39 *GBA* (N370S) PD patients in the Parkinson’s Progression Markers Initiative (PPMI) study, we estimated the temporal trajectories of putaminal-specific binding ratios using an integrating function between baseline values and their annual change rates. In order to test reproducibility, we computed another trajectory for sPD using positron emission tomography data of 38 sPD patients at Gangnam Severance Hospital (GSH). Temporal trajectories of sPD were compared between the groups separated by age at onset (AAO) and polygenic load for common PD risk variants, and also compared with genetic PD. sPD patients in both the PPMI and GSH cohorts showed similar onset of dopaminergic degeneration around 10 years before motor onset. Early-onset PD patients exhibited later onset of degeneration and a faster decline in dopaminergic activity during the premotor period than late-onset patients. sPD patients with high polygenic load were associated with earlier onset and slower progression of dopaminergic dysfunction. Compared to the sPD and *LRRK2* PD groups, *GBA* PD patients exhibited faster deterioration of dopaminergic function during the premotor stage. Dopaminergic dysfunction in PD appears to start about 10 years before motor onset. Genetic factors may be contributing to the heterogeneity of dopaminergic deterioration during the premotor stage.

## Introduction

Disease-modifying therapies are critically needed for the effective treatment of Parkinson disease (PD). Such interventions may be best suited to populations at high risk before the manifestation of parkinsonian motor symptoms. In a recent study of hyposmic subjects, participants with reduced dopamine transporter (DAT) levels in ^123^I-N-3-fluoropropyl-2-β-carboxymethoxy-3β-(4-iodophenyl) nortropane (^123^I-FP-CIT) single-photon emission computed tomography (SPECT) were found to have a higher risk for phenoconversion to PD^1^. In addition, DAT availability in SPECT scans is known to correlate with synaptic dopaminergic deficit and degeneration in nigral dopaminergic neurons^2^. Therefore, establishing the trajectory of dopaminergic dysfunction during the premotor period is essential for proper design and interpretation of disease-modifying interventions in pre-manifesting population.

However, previous statistical estimations for striatal dopaminergic dysfunction during the premotor period have been inconsistent^3–6^. Moreover, although the genetic background of individual patients is associated with various clinical features of PD such as age at onset (AAO) and non-motor features in the premotor stage^7–10^, little is known about the genetic impact on dopaminergic dysfunction in the premotor period. Due to practical limitations^11^, it is difficult to compose a premotor cohort with an adequate sample size. Under these circumstances, a mathematical approach such as ordinary differential equation modeling represents an alternative approach to predicting a temporal trajectory for dopaminergic dysfunction.

In the present study, we estimated the temporal trajectory of putaminal dopaminergic dysfunction during the premotor period by integrating the function between baseline and annual change rates using longitudinal ^123^I-FP-CIT SPECT data from the Parkinson’s Progression Markers Initiative (PPMI) study^12,13^. The estimated trajectory was compared with an independent cohort (Gangnam Severance Hospital (GSH), Seoul, South Korea) who underwent ^18^F-FP-CIT positron emission tomography (PET). We also investigated the effect of AAO and polygenic burden on the trajectories. In addition, the trajectory of DAT availability in PD patients with *GBA* or *LRRK2* mutation were estimated.

## Results

### Demographics

**Fig. 1:**
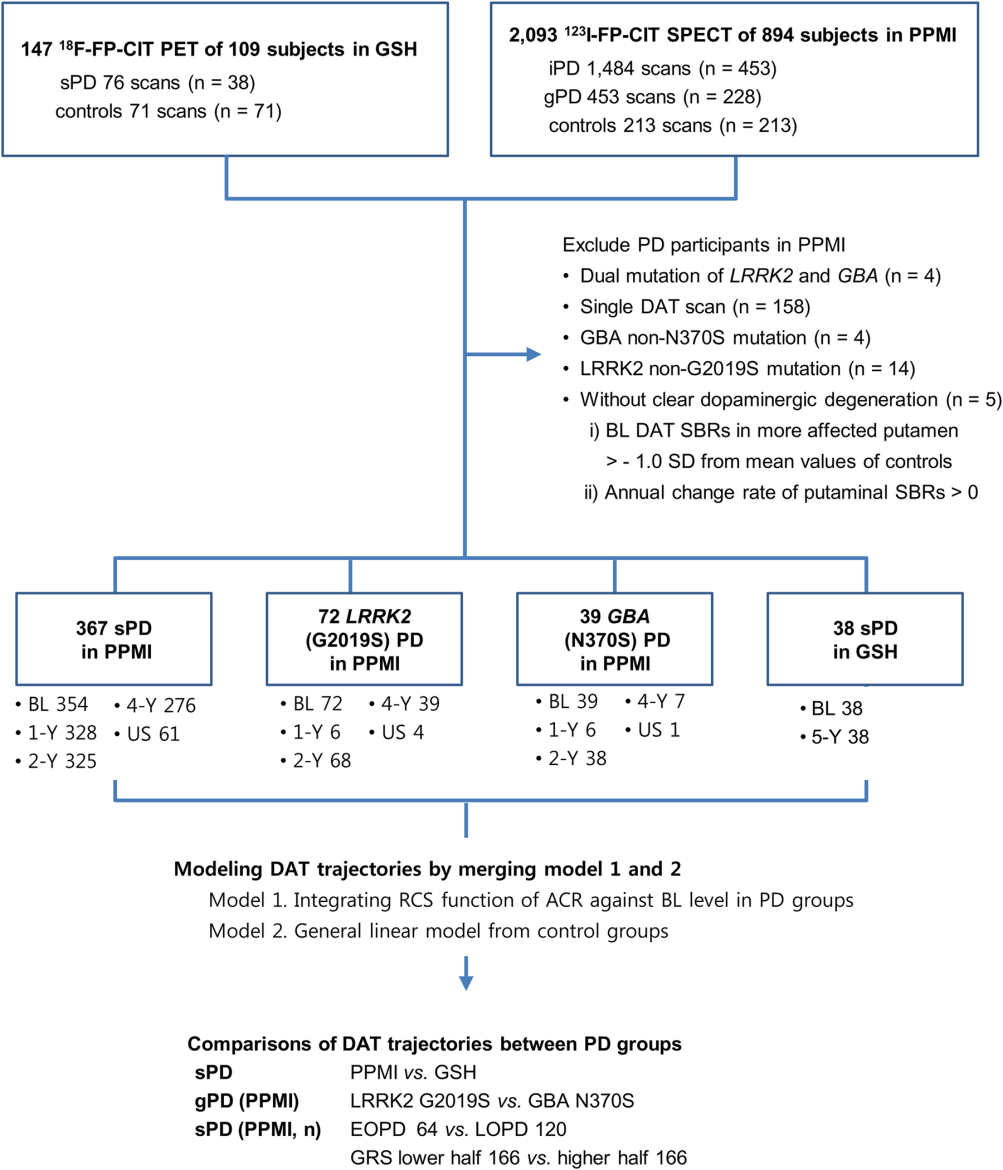
Study design. GSH = Gangnam Severance Hospital cohort; PPMI = Parkinson’s Progression Markers Initiative; PET = positron emission tomography; SPECT = single-photon emission computed tomography; iPD = idiopathic PD cohort in PPMI study; sPD = sporadic PD cases; gPD = genetic PD cohort in PPMI study; DAT = dopamine transporter; SBR = specific binding ratio; SC = screening visit in each cohort; BL = baseline; 1, 2, 4, 5-Y = 1, 2, 4, 5-year follow-up visit; US = unscheduled visit; F/U = follow-up; RCS = restricted cubic spline; ACR = annual change rate; AAO = age at onset; GRS = genetic risk score; *comparison within the PPMI database.

Demographic characteristics of the included PD and control subjects are summarized in Table 1. In the PPMI cohort, 367 sporadic PD (sPD), 72 *LRRK2* (G2019S) PD, 39 *GBA* (N370S) PD patients, and 213 controls were analyzed, and 38 sPD patients and 71 controls in the GSH cohort were investigated (see Fig. 1). sPD patients in the PPMI and GSH cohorts had similar age, disease duration and AAO at baseline. However, the sPD group in the PPMI study showed a male predominance and lower Movement Disorder Society-sponsored Unified Parkinson’s Disease Rating Scale (MDS-UPDRS III) total scores at baseline (generalized linear model (GLM), *p* = 0.008) compared to the GSH cohort. In addition, the *LRRK2* PD group had female predominance when compared to the sPD patients and controls in the PPMI study. Despite a considerable overlap, sPD patients showed significantly higher genetic risk score (GRS) than controls (independent *t*-test, *p* < 0.001). The early-onset PD (EOPD) group had significantly higher GRS than late-onset PD (LOPD) patients (EOPD 6.371 ± 0.609 vs. LOPD 6.068 ± 0.668; independent *t*-test, *p* = 0.004), while *LRRK2* and *GBA* PD patients had significantly longer disease duration than the sPD group (Mann-Whitney *U* test, *p* < 0.001), but AAO did not show a significant difference between the two genetic PD (gPD) groups. *GBA* PD subjects had higher MDS-UPDRS III scores than the sPD and *LRRK2* PD patients.

**Table 1:** Demographics and characteristics of the participants.

	PPMI cohort				GSH cohort	
	Controls	sPD	*LRRK2*	*GBA*	Controls	sPD
	(*n* = 213)	(*n* = 367)	(*n* = 72)	(*n* = 39)	(*n* = 71)	(*n* = 38)
Age (years)	60.9 ± 11.3	60.9 ± 11.3	62.0 ± 8.6	61.5 ± 11.2	59.7 ± 10.3	62.3 ± 9.7
Female subjects	73 (34.3%)	127 (34.6%)	38 (52.8%)*^†^	17 (43.6%)	39 (54.9%)	20 (52.6%)
AAO (years)	n.a.	60.7 [13.6]	59.8 [11.3]	57.6 [15.4]	n.a.	59.7 [16.1]
Disease duration (years)	n.a.	1.5 [1.6]	3.5 [3.3]^†^	3.3[4.2]^†^	n.a.	1.1 [1.3]
MDS-UPDRS III	n.a.	20.8 ± 8.7	20.9 ± 9.2	26.6 ± 11.2^†‡^	n.a.	24.9 ± 9.1
H&Y stage (I/II/III)(%)	n.a.	163/204/0 (44.4/55.6/0)	11/26/4^†^ (26.8/63.4/9.8)	11/20/1^†^ (34.4/62.5/3.1)	n.a.	n.a.
GRS	5.90 ± 0.63	6.19 ± 0.65*	n.a	n.a	n.a.	n.a.
Striatal SBRs						
CN, more affected	2.97 ± 0.61 *(*averaged*)*	1.83 ± 0.53*	1.78 ± 0.50*	1.70 ± 0.72*	5.84 ± 1.24 *(*averaged*)*	4.35 ± 1.30*
CN, less affected	2.97 ± 0.61 *(*averaged*)*	2.14 ± 0.59*	1.96 ± 0.59*^†^	1.94 ± 0.75*^†^	5.84 ± 1.24 *(*averaged*)*	4.76 ± 1.39*
PUT, more affected	2.13 ± 0.55 *(*averaged*)*	0.66 ± 0.24*	0.63 ± 0.21*	0.67 ± 0.43*	8.07 ± 1.27 *(*averaged*)*	3.75 ± 1.07*
PUT, less affected	2.13 ± 0.55 *(*averaged*)*	0.98 ± 0.37*	0.86 ± 0.30*^†^	0.89 ± 0.49*	8.07 ± 1.27 *(*averaged*)*	4.80 ± 1.22*

Mean ± SD for variables with Gaussian distribution. Median [interquartile range] for variables showing non-Gaussian distribution. Abbreviations: *PPMI* Parkinson’s Progression Markers Initiative, *GSH* Gangnam Severance Hospital, *AAO* age at onset, *MDS-UPDRS III* Movement Disorder Society sponsored Unified Parkinson’s Disease Rating Scales part III, total scores, *GRS* genetic risk scores, *SBRs* specific binding ratios, *CN* caudate nucleus, *PUT* putamen, *significant difference with control subjects, ^†^significant difference with sPD group, ^‡^significant difference between GBA and LRRK2 group, *n.a.* not available.

In both cohorts, striatal-specific binding ratios (SBRs) for DAT imaging were significantly lower in the PD patients than controls. Compared to the sPD group, *LRRK2* PD patients showed lower SBRs in the less-affected caudate nucleus and putamen, and *GBA* PD exhibited lower SBRs in the less-affected caudate nucleus.

### Trajectories of putaminal SBR in sPD patients

**Fig. 2:**
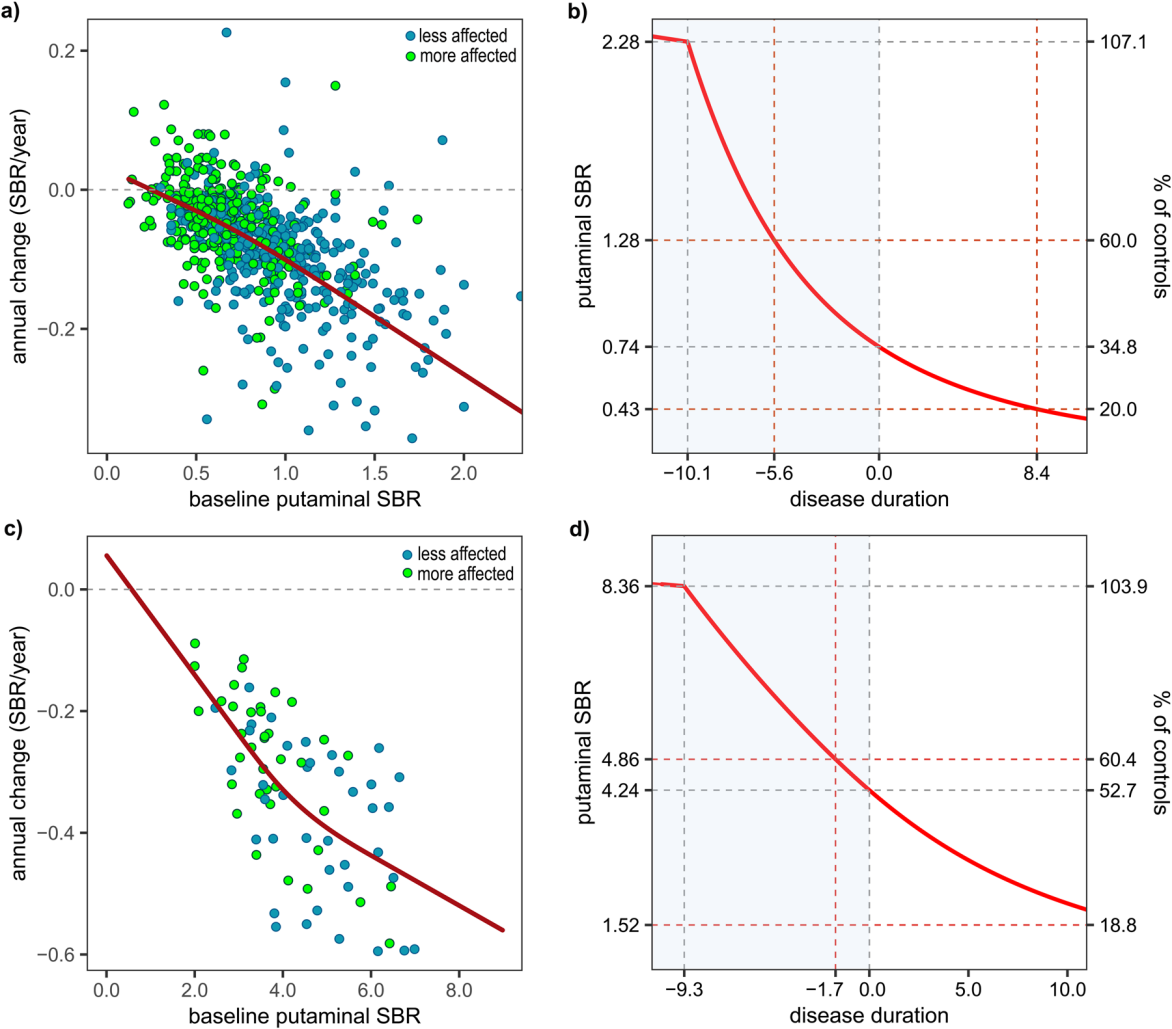
Annual changes in putaminal DAT SBRs as a function of baseline levels and estimated temporal trajectories in sPD patients. Fitted curves on the scatter plots were obtained with the RCS model in PPMI (**a**) (^123^I-FP-CIT SPECT) and GSH (**c**) (^18^F-FP-CIT PET) cohorts. Outliers beyond the first or third quartile ± 3 × interquartile range (5 putamen, sPD group of PPMI cohort) are not shown. All outliers were included in the calculation of temporal trajectories. Temporal trajectories in the PPMI (**b**) and GSH (**d**) cohorts were acquired with a modified Euler’s method for solving the first-order differential equation.

In the restricted cubic spline (RCS) curves in sPD patients from the PPMI and GSH cohort, almost linear decrements were observed between the annual change rate and baseline SBRs, suggesting that changes in DAT levels would show a negative exponential pattern (Fig. 2a, c). Estimated temporal trajectories of the putaminal SBRs showed that dopaminergic degeneration started at a similar time point in both cohorts: 10.1 years before motor onset in the PPMI cohort and 9.3 years prior in the GSH cohort. However, the sPD patients in the PPMI cohort showed greater dopaminergic deficits at the onset of motor symptoms (putaminal SBR = 34.8% vs. 52.7% of controls) and more rapid reduction in SBRs than those in the GSH cohort (Fig. 2b, d). The additional analysis for posterior putaminal SBRs of the GSH cohort exhibited a similar pattern of DAT decline to the sPD group in PPMI (Supplementary Fig. 1).

### Comparison between EOPD and LOPD

**Fig. 3:**
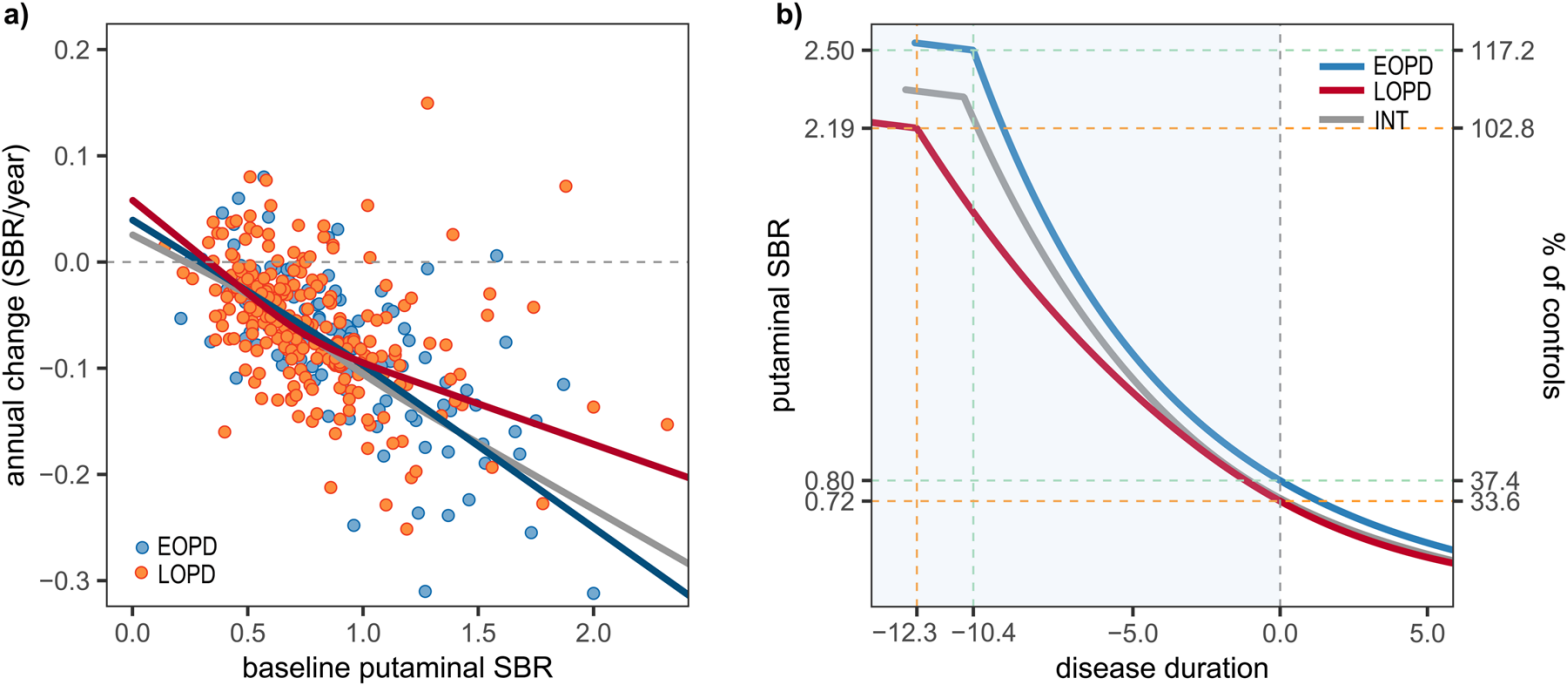
Comparison of annual change rates against baseline SBRs and DAT trajectories between EOPD, intermediate-onset, and LOPD groups in the PPMI cohort. **a** sPD patients with early and intermediate onset showed similar curves, but annual change rates in EOPD patients are lower within the range over 0.9 of the baseline SBR compared to the LOPD group. Outliers beyond the first or third quartile ± 3 × interquartile range were not shown (2 putamen, sPD group of PPMI cohort). All outliers were included in the calculation of temporal trajectories. **b** Estimated trajectory of the intermediate-onset group was close to EOPD group, however, EOPD patients show a shorter premotor phase and faster DAT decline during the premotor period than the LOPD group (B; INT = intermediate onset).

In the linear mixed effects model (LMM) test with the interaction term, AAO was associated with lower annual change rates (faster decline), however, it did not reach statistical significance (estimate of AAO −0.0012, SE 0.0007, *p* = 0.087; Supplementary Table 1). Interaction between AAO and baseline SBRs exhibited a significant positive effect on annual change rates of DAT SBRs, showing that an older AAO was associated with high annual change rates (slow decline) along with an increase in DAT SBRs (estimate of interaction term 0.0015, SE 0.0007, *p* = 0.033; Supplementary Table 1). In the RCS fits, sPD patients with early and intermediate onset showed similar curves, although annual change rates in patients with intermediate onset became slightly higher along with an increase in baseline SBRs. Compared to the LOPD group, EOPD patients showed a greater annual change in the baseline SBR range over 0.9, suggesting a faster decline in dopaminergic input at the early stage of dopaminergic degeneration (Fig. 3a). Calculated SBRs at motor onset were higher in the EOPD (median [interquartile range]; 0.75 [0.34]) patients than LOPD (0.67 [0.29]) although the difference did not reach statistical significance (Mann-Whitney *U* test, *p* = 0.060). In the estimated trajectories, the DAT trajectory of EOPD was close to that of patients with intermediate onset. The subset with intermediate onset showed a slightly slower decline in SBRs after the onset of dopaminergic dysfunction, and had a similar premotor period (EOPD 10.4, intermediate group 10.7 years). In contrast, the LOPD group had a longer premotor period (12.3 years) and faster decline in SBRs compared to EOPD patients (Fig. 3b; SBR reduction during premotor stage, EOPD 0.16/year vs. LOPD 0.12/year).

### Effect of genetic factors on the trajectories of putaminal SBR

**Fig. 4:**
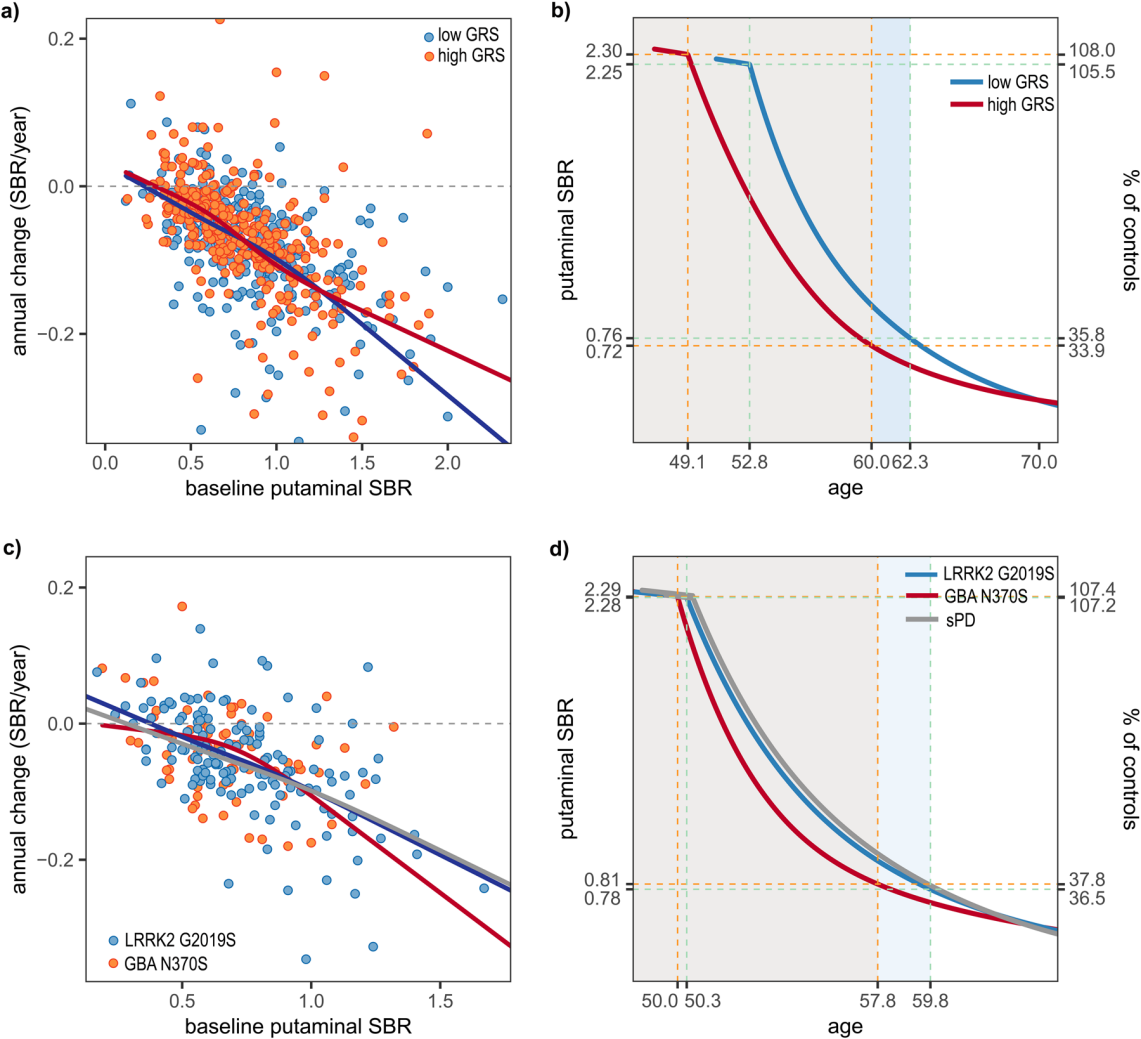
Comparison of annual change rates against baseline SBRs and DAT trajectories according to genetic aspect. Outliers beyond the first or third quartile ± 3 × interquartile range are not shown in panel (**a**) (5 putamen, sPD group of PPMI cohort). All outliers were included in the calculation of temporal trajectories. The sPD patients with high GRS values have a smaller annual change rate (**a**) and longer premotor phase in the estimated trajectory than those with low GRS (**b**). Compared to *LRRK2* PD patients, *GBA* PD participants exhibit a lower annual change rate within the range over 0.9 of the baseline DAT SBR (**c**), and a rapid decline in estimated DAT SBRs during premotor phase (**d**).

In sPD patients of Caucasian ancestry, a comparison of annual change rates using the LMM test did not show a significant difference between the high- and low-GRS groups (Supplementary Table 1). However, GRSs showed a trend for correlation with a smaller annual change of putaminal SBRs (LMM, estimate = 0.012, SE 0.006, *p* = 0.052). When sPD patients in the first (<25% quartile) and fourth quartile (>75 percentile) of GRSs were compared by LMM test, patients in the fourth quartile of GRSs showed smaller annual change rates (estimate = 0.023, SE = 0.012, *p* = 0.049). In comparisons of the RCS fits, the high-GRS group showed less annual change in the baseline SBR range over 1.2 than low-GRS patients (Fig. 4a). GRSs in the patients were associated with early AAO (Pearson’s correlation test, ρ = −0.136, *p* = 0.013), suggesting a leftward shift in estimated trajectory for the high-GRS group. In addition, GRSs exhibited a significant negative correlation with SBRs at motor onset (Pearson’s correlation test, ρ = −0.115, *p* = 0.036). As a result, patients with high GRS showed a longer premotor phase (high vs. low GRS, −10.9 vs. −9.5 years), and a slightly slower decline in putaminal dopaminergic activity (Fig. 4b; SBR reduction rate during premotor phase, and low GRS 0.16/year vs. high GRS 0.14/year). When sPD patients were categorized by GRS quartiles, higher GRS quartiles were associated with earlier onset of DAT decline and a slower SBR reduction rate during the premotor stage (Supplementary Fig. 2).

*GBA* PD patients showed smaller mean values for annual SBR decline than the sPD group (LMM, effect of *GBA* PD groups; estimate = 0.0637, SE = 0.0205, corrected *p* for multiple testing = 0.006), however, annual changes in the *GBA* PD subset became greater along with an increase in baseline SBRs (LMM, interaction between baseline SBRs and groups; estimate = −0.0917, SE = 0.0217, corrected *p* for multiple testing < 0.001; Supplementary Table 1 and Fig. 4c). In comparisons between the sPD and *LRRK2* PD groups, the main effect of groups and interaction terms did not show significant differences. Compared to *LRRK2* PD patients, *GBA* PD patients showed greater annual changes in the baseline SBR range over 0.9 (LMM; interaction between baseline SBRs and group; estimate = −0.0652, SE = 0.0297, nominal *p* = 0.030; Fig. 4c and Supplementary Table 1), however, the difference became no more significant after correction for multiple testing. In the estimated trajectories, onsets of DAT decline in the *GBA* and *LRRK2* PD groups started at a similar age (*GBA* PD 50.0 vs. *LRRK2* PD 50.3 years), however, *GBA* PD patients showed a rapid decrease in estimated SBRs and earlier onset of motor symptom compared to the *LRRK2* PD and sPD groups (SBR reduction during premotor phase; *GBA* PD 0.19/year, LRRK2 PD 0.16/year, sPD 0.15/year). The estimated trajectory for *LRRK2* PD exhibited a close pattern with sPD (Fig. 4d).

## Discussion

In this study, we modeled a temporal trajectory for putaminal dopaminergic deficit during the premotor period in PD patients using extensive longitudinal PPMI data. The premotor phase was estimated to be about 10 years (Fig. 2b), and the trajectory using different imaging modality in another cohort exhibited a similar result (Fig. 2d). Younger AAO was associated with a shorter premotor phase and faster dopaminergic degeneration (Fig. 3a, b and Supplementary Table 1). In addition, sPD patients with higher polygenic load exhibited an earlier onset of dopaminergic degeneration and motor symptoms, compared to those with less PD-risk SNPs (Fig. 4a, b). Finally, onset ages of DAT decline were similar between *GBA* PD, *LRRK2* PD, and sPD groups in the present study, however, patients with the *GBA* N370S variant had more rapid deterioration of putaminal dopaminergic function during the premotor phase (Fig. 4c, d and Supplementary Table 1). We found that individual genetic background may influence the premotor trajectory of striatal dopaminergic degeneration in PD.

A postmortem study counting nigral neurons and several ^18^F-DOPA PET studies estimated 5.0–7.0 years for the premotor phase in PD patients^4–6,14^. However, axonal degeneration and synaptic dysfunction precede the death of nigral neurons in PD^15^, and the activity of presynaptic aromatic amino acid decarboxylase is up-regulated for compensation in the premotor phase^16,17^. Therefore, previous studies may have underestimated the premotor phase^3^. Moreover, several studies assumed a linear decline for dopaminergic input^4,5,14^. A PET study assessing vesicular monoamine transporter 2 (VMAT2) availability estimated the premotor phase to be about 10 years in PD patients with an AAO of 70 years by fitting a negative exponential curve^3^. Even without a priori assumptions for a curved shape, our trajectory models obtained from two different cohorts with different imaging modalities exhibited a negative exponential pattern, and the estimated premotor phase was about 10 years as with the previous study (Fig. 2b, d).

In contrast to our analyses (Fig. 3b and Supplementary Table 1), a longitudinal PET study for VMAT2 availability estimated that PD patients with younger AAO may have prolonged dopaminergic degeneration in the premotor stage, and showed a lower radiotracer uptake and slower reduction rate in the motor phase^3^. A ^99m^Tc-TRODAT-1 study with a small sample size (*n* = 14) reported lower DAT availability in EOPD than LOPD patients^18^. However, subsequent neuroimaging studies have shown inconsistent results^19,20^. In a cross-sectional study of PPMI data, EOPD patients were found to be less likely to show less motor dysfunction and less striatal dopaminergic deficit than their disease duration-matched LOPD counterparts^21^. Moreover, unlike our method without an assumption for a curved shape, the previous study fitted plots to a negative exponential function, assuming that a fixed proportion of dopaminergic activity decreased over time^3^.

It has been postulated that down-regulation of DAT expression may compensate for the decreased dopaminergic activity and maintain basal ganglia output within normal limits^2^. EOPD patients are expected to have more efficient compensatory mechanisms for dopaminergic deficit than LOPD patients^3,20^. Therefore, it is possible that a rapid decline in SBRs during the premotor period in our EOPD trajectory might reflect a compensatory down-regulation of DAT expression. EOPD patients have less severe parkinsonian motor deficits^21^, and the duration from the onset to reach H&Y stage 3 is longer in EOPD than LOPD patients^22^. As estimated in our analyses, favorable outcomes for EOPD patients might be associated with a compensatory mechanism and relatively preserved dopaminergic activity at baseline compared to LOPD patients.

In our previous analysis of the PPMI database, a GRS composed of common PD risk variants was associated with a slower progression of dopaminergic degeneration (Fig. 4a, b)^7^. High polygenic burden in PD is associated with early onset of motor signs^7,8^, and so we have therefore speculated that earlier onset of dopaminergic dysfunction may be related to a slower decrease in DAT levels^7^. Indeed, sPD patients with high GRS in the present study showed a slower decline in the estimated SBRs, suggesting that the summarized effect of common PD risk variants may not be related to acceleration of dopaminergic degeneration. However, recent genome-wide association studies (GWASs) have demonstrated that PD risk variants were not associated with progression of parkinsonian motor deficits^23^, and several well-established PD risk loci did not significantly correlate with AAO in PD patients^24^. EOPD patients in the present study showed a rapid decrease in DAT availability despite higher GRSs than the LOPD group. These findings suggest that genetic factors not related to disease manifestation may have an impact on the progression of dopaminergic dysfunction. Further studies addressing the genetic background of dopaminergic deterioration in PD will be required.

In contrast to our results, a recent cross-sectional analysis of the PPMI cohort reported higher putaminal ^123^I-FP-CIT SBRs in PD patients with monogenic mutations than the sPD group^25^. This study did not exclude PD patients with putaminal SBRs close to the mean of the controls, and thereby the ranges of putaminal SBRs were reported to be 0.19–2.45 in *GBA* PD and 0.17–2.27 in the *LRRK2* PD patients. Given the putaminal SBRs in the control group (mean ± SD, 2.13 ± 0.55), the previous study might have included participants with genetic mutations but with no clear striatal dopaminergic deficits. Therefore, caution is needed when interpreting whether gPD patients at early-stage PD may have more preserved dopaminergic activity compared to the sPD group. In addition, we hypothesized that non-manifesting *GBA* N370S carriers may have similar DAT levels to healthy controls. However, a recent report by PPMI investigators showed higher putaminal DAT SBRs for unaffected *GBA* carriers than the controls^9^. Unfortunately, DAT SBRs for the unaffected genetic groups are not publically available, thus it is possible that the estimated premotor period for *GBA* PD patients in the present study could be underestimated (Fig. 4d).

PD patients with *GBA* mutations are known to show malignant disease progression when compared to sPD, however, N370S is associated with less severe clinical presentations than other *GBA* variants^26,27^. In a meta-analysis of 12 clinical studies, N370S was not associated with progression of parkinsonian motor deficits^28^. A neuroimaging study showed similar dopaminergic deficits between PD patients with the *GBA* N370S mutation and without, although the sample size was small (*GBA* N370S, *n* = 8)^26^. In contrast, a recent cross-sectional analysis of the PPMI study reported higher MDS-UPDRS III scores in unaffected *GBA* mutation carriers^9^. A longitudinal study observed that non-manifesting carriers with *GBA* N370S showed rapid deterioration of parkinsonian motor deficits^29^, which is a good predictor of dopaminergic neuronal loss^30^. Therefore, as shown in our estimation (Fig. 4d and Supplementary Table 1), it is possible that PD patients with the *GBA* N370S mutation may experience rapid progression of striatal dopaminergic dysfunction during the premotor stage, and consequently, the *GBA* N370S variant may be associated with disease manifestation at a younger age and earlier diagnosis of PD compared to sporadic cases^31^. *GBA* N370S carriers could therefore require screening for PD at a young age and early trials of possible disease-modifying therapies.

In a longitudinal study including non-manifesting *LRRK2* G2019S carriers, the annual decline rate of ^123^I-FP-CIT binding did not show a significant difference between PD converters and non-converters^32^, suggesting that dopaminergic degeneration in *LRRK2* carriers precedes the parkinsonian motor symptoms. In that study, PD converters exhibited about a 5% annual decline rate, which is a lower number within the range of the previously reported decline rate (4–10%/year) in sPD patients^32^. Therefore, as shown in our estimated trajectory, *LRRK2* PD patients may have a similar^33,34^ or slightly slower dopaminergic deterioration compared to sPD. *LRRK2* mutations are associated with a variable degree of Lewy body burden^35^, and individuals with *LRRK2* G2019S may have non-Lewy body pathology, such as progressive supranuclear palsy, corticobasal degeneration, or amyotrophic lateral sclerosis^36,37^. However, a similar pattern of DAT decline in the sPD group suggests that *LRRK2* G2019S carriers may be good candidates for investigating striatal dopaminergic dysfunction and nervous adaptation in the premotor stage of PD^32,38^. Meanwhile, *LRRK2* PD patients show a relatively milder presentation and slower progression of parkinsonian motor deficits than sPD cases^39^. Given our estimated trajectories, less severe parkinsonian motor deficits in *LRRK2* PD patients might reflect a possible compensation by down-regulation of DAT expression or other non-dopaminergic mechanism^2^.

We note several limitations to our study findings. First, we estimated the trajectory models of putaminal SBR for about 20 years using longitudinal data obtained for a limited time window (<5 years). This limitation is not specific to our study design, but common for all previous studies predicting the hidden trajectory. Second, our study has no a priori assumption for the shape of the trajectory, therefore we are unable to test a statistical significance for estimated SBRs or adjust for the potential influence of clinical factors. Our results are required to be cross-validated by further longitudinal studies including prodromal PD patients. Third, due to limitations of the dataset, we were unable to investigate a potential effect of dopaminergic medication on the decline of DAT SBRs. The PPMI database does not provide dosage information for the antiparkinsonian medication taken by each PD subject. However, several studies in animal PD models and patients have suggested that chronic dopaminergic stimulation using levodopa or pramipexole may not cause significant changes in striatal DAT uptake^40–43^. Fourth, longer disease duration and low putaminal SBRs of the less-affected side in monogenic PD patients may cause a deviation in the RCS curve and eventual skew of the trajectory. However, our LMM tests covariated with disease duration also showed significant differences in annual SBR changes between the *GBA* PD, *LRRK2* PD, and sPD groups. In addition, we included only *GBA* N370S or *LRRK2* G2019S mutations in assessment of the homogeneous patient groups. However, there is considerable heterogeneity in phenotypes between *GBA* mutations (e.g., L444P or 84GG). Therefore, further studies for *GBA* PD patients with non-N370S mutations will be required. Finally, we included sPD cases from the GSH to reproduce the temporal trajectory of those in the PPMI study, however, the GSH cohort had a small sample size and employed ^18^F-FP-CIT PET. Differences in sample size, disease severity, and imaging modality may cause discordance in the DAT decline rates between groups.

In summary, putaminal dopaminergic deficits may start around 10 years before the motor onset, and so a screening test using DAT scans may be helpful for identifying prodromal PD patients in high-risk populations with age >50 years. However, our results suggest that the onset and progression of DAT decline may vary depending on individual genetic background. Patients with *LRRK2* G2019S showed a similar trajectory of DAT decline to the sPD group, whereas *GBA* PD patients exhibited a rapid decrease in DAT availability during the premotor phase compared to sPD or a subset with the *LRRK2* mutation. sPD patients with high polygenic load are associated with an earlier onset but a trend for slower progression of dopaminergic degeneration. In contrast, EOPD patients in the present study are associated with a shorter premotor period and faster decline of estimated DAT levels than the LOPD group, suggesting that non-PD risk genetic factors may have a substantial effect on AAO and dopaminergic deterioration. Although investigation in further studies is required, our findings may be of relevance for the planning and interpretation of disease-modifying trials of PD.

## Methods

### Participants

A brief summary of the study design is shown in Fig. 1. We analyzed data from PD patients and healthy controls in two separate cohorts.

First, we used anonymized and de-identified clinical data in the PPMI database as of Feb 2020. The currently ongoing PPMI study aims to identify biomarkers reflecting the progression of PD, with detailed inclusion criteria for the sporadic and gPD patients described in previous literature^7,25^. In the PPMI protocol, PD patients were scheduled to undergo ^123^I-FP-CIT SPECT at baseline and at the 1-, 2-, and 4-year follow-up visits. Using the idiopathic PD and gPD cohorts in the PPMI database, we included only those PD patients who underwent ^123^I-FP-CIT scans at least twice and then dichotomized the PD patients into sporadic PD (sPD) and genetic PD (gPD) carrying *GBA* or *LRRK2* mutations. The “Idiopathic PD” group in the PPMI study included some PD patients with *GBA* (*n* = 11) and *LRRK2* mutations (*n* = 6). These subsets were classified as *GBA*- or *LRRK2*- PD groups in the present study. The excluded PD subjects had older age at baseline and longer disease duration, however, other characteristics including striatal DAT SBRs at baseline were not significantly different from those included in the analyses (Supplementary Table 2). Healthy controls with ^123^I-FP-CIT SPECT scans were also included to estimate the onset of DAT decline.

Second, we included sporadic drug-naive PD patients who met the Movement Disorder Society revised clinical diagnostic criteria for PD^44^ and completed two ^18^F-FP-CIT PET scans at GSH (Seoul, South Korea) for clinical purposes at the time of the first diagnosis and after 5.2 ± 1.4-year follow-up. In addition, we included healthy controls who underwent ^18^F-FP-CIT PET scans.

We classified sPD patients from the PPMI cohort into early-onset (EOPD, AAO < 50 years), intermediate-onset, and late-onset PD (LOPD, AAO ≥ 65 years) groups^22^, and compared the progression of putaminal dopaminergic dysfunction. In the PPMI study, the Movement Society sponsored Unified Parkinson Disease Rating Scale (MDS-UPDRS) part III score and Hoehn & Yahr (H&Y) stage were measured in all PD patients. Classic UPDRS motor scores for the GSH cohort were converted into MDS-UPDRS III using published formula^45^.

### Ethical approval

The PPMI study is registered at ClinicalTrials.gov (NCT01141023). Each PPMI site received approval from an ethics committee on human experimentation prior to study initiation. The Institutional Review Board of Gangnam Severance Hospital also approved the present study for cross-validation. Written informed consent for the research was obtained from all individuals participating in the PPMI study and GSH cohort.

### Genetic testing and calculation of GRSs

In the PPMI study, sPD patients and healthy controls underwent genotyping for single nucleotide polymorphisms (SNPs), and the gPD patients with predetermined *LRRK2* or *GBA* mutations were also included. Detailed methods for the genetic testing are described at www.ppmi-info.org. Eight PD patients with dual mutations of *LRRK2* and *GBA* were excluded. To compose homogeneous patient groups, we included only *LRRK2* G2019 or *GBA* N370S mutations in the gPD subsets. To estimate the effect of polygenic load on long-term change in dopaminergic dysfunction in sPD patients, we calculated GRSs using 45 common PD risk SNPs in 152 controls and 331 sPD. In order to consider participants’ ethnicity, we undertook a principal component (PC) analysis using HapMap genetic data^46,47^ and NEUROX chip data of PPMI. After merging PPMI and HapMap data, we extracted 20 leading PCs with the “-pca” option in PLINK^46^. Using two leading PCs, we classified ethnicity of sPD participants, and finally included patients who were close to HAPMAP participants with European ancestry (Supplementary Fig. 3). The GRS was defined as the number of risk alleles weighted by log odds ratios (ORs). The ORs for PD risk SNPs were obtained from a public database (www.pdgene.org), which referred meta-ORs from previous meta-analysis of PD GWASs including 5353 European ancestry PD and 5551 control subjects (Supplementary Table 3)^48^. Therefore, the weights were ascertained independently from the database of PPMI.

### ^123^I-FP-CIT SPECT and ^18^F-FP-CIT PET scan and image processing

For the PPMI cohort, SPECT images were acquired at 3–4 h after injecting ^123^I-FP-CIT and reconstructed with iterative method. In PMOD (PMOD Technologies, Zurich, Switzerland), reconstructed images were corrected by attenuation, smoothed with 6 mm full-width at half-maximum (FWHM) of Gaussian filter, and finally normalized to the Montreal Neurologic Institute (MNI) space. Eight consecutive axial slices around the slice with highest striatal uptake were averaged to create single axial slice, and then regions-of-interest (ROIs) for each side of the caudate and putamen were applied to measure regional uptake. Additionally, a ROI for the occipital cortex was applied to measure an uptake value for reference region.

For the GSH cohort, PET images were acquired in a Biograph 40 TruePoint PET/CT scanner (Siemens Medical Solutions; Malvern, PA, USA) at 3 h after injecting ^18^F-FP-CIT for 10 min. After the correction of attenuation with computed tomography images acquired before emission scan, 3D PET images within a 512 × 512 × 110 matrix with voxel size 0.668 × 0.668 × 2 mm) were reconstructed with iterative method. In 3.0 Tesla scanner (Signa EXCITE, GE Medical Systems, Milwaukee, WI, USA), axial T1-weighted brain MR images were also acquired with 3D-spoiled gradient-recalled sequences (repetition time = 6.8 ms, echo time = 1.6–11.0 ms, flip angle = 20°, 512 × 512 matrix, voxel size 0.469 × 0.469 × 1 mm). For the image processing, statistical parametric mapping 12 (SPM12; Wellcome Trust Centre for Neuroimaging, London, UK) and in-house software implemented in MATLAB 2017b (MathWorks, Natick, MA). PET images were first coregistered to MR images. By using the diffeomorphic anatomical registration through exponentiated lie algebra (DARTEL) tool and in-house DARTEL template, MR images were spatially normalized to MNI space. Subsequently, the PET images were also normalized by applying the flow field normalizing MR images. By overlaying in-house FreeSurfer-based atlas for DARTEL^49^, we measured regional uptake values for each side of the caudate and putamen. SBRs of posterior putamen also were calculated. The uptake in the cerebellar cortex was used for the reference region.

SBRs were calculated as follows: SBR = (target − reference)/reference. We used putaminal SBRs to estimate the long-term trajectory. To avoid a bias from errors in the diagnosis or image processing, we excluded PD subjects (*n* = 5) in the PPMI cohort who satisfied both (i) showing baseline putaminal SBRs over −1.0 SD from the mean of controls and (ii) annual change rate of putaminal DAT level >0.

### Estimating the trajectory of putaminal SBR during the premotor stage

By using both more- and less-affected putaminal SBRs, we composed two models of SBR decline: the non-PD state before the onset of dopaminergic degeneration and the PD state after onset. For the non-PD state, we hypothesized that dopaminergic function in PD subjects would be identical to normal control group before initiation of their dopaminergic degeneration. Using both averaged putaminal DAT SBRs as dependent variable and age as independent variable, the linear regression model was obtained (averaged putaminal SBRs ~*C*_1_ × age + *C*_2_). Dopaminergic function before onset of deterioration was estimated by applying the linear model for PD subjects.

For the PD-state model, the modified Euler method was employed to calculate the temporal trajectory after the onset of DAT decline^12,13^. Under the hypothesis that values of individual short-term time series can be placed along a continuous, ordered measure of disease process, ordinary differential equation modeling such as modified Euler method allows to figure out a temporal trajectory of values under a given differential equation (Supplementary Fig. 4). To obtain an outcome of differential equation, we first applied a linear regression model for each putaminal SBR to calculate the annual change rates, and then plotted annual changes in SBRs against baseline values. To avoid potential bias from repeated measurements, we employed a mixed effect model with cubic regression splines using baseline SBRs as the predictor, and annual SBR change as the responder variable^50^. We then applied a RCS function between the baseline SBRs and predicted annual change rates from the mixed effect model with the cubic regression splines. The knots in the mixed effect model and RCS function were placed at 5-, 35-, 65-, and 95-percentile values of baseline SBRs^12,13,51^. For groups with sample sizes (count of putamen) <100, we placed knots at 10-, 50-, and 90-percentile values (Supplementary Fig. 5). Estimated putaminal SBRs as a function of time in the year were obtained using the modified Euler’s method for solving the first-order differential equation. To anchor trajectories along with disease duration, we calculated putaminal SBRs of the more-affected side at disease duration = 0 from the aforementioned individual linear regression model, and the mean of the estimated SBR at motor onset was obtained to find the time point for the onset in the trajectory model. The median for the AAOs in each PD group was added to disease duration when the time axis was converted into age (Supplementary Fig. 6). Finally, we considered the onset of striatal dopaminergic dysfunction as the intersection point between non-PD and PD-state models^3^ (Supplementary Fig. 7). In this manuscript, *premotor phase* refers to an interval between the beginning of dopaminergic degeneration and the onset of motor symptoms.

### Statistical analyses

We used R software (version 3.6.1; r-project.org) for all statistical analysis and modeling of temporal trajectories. Independent *t*-test and Chi-square test were used for the group comparisons of continuous and categorical variables, respectively. Comparisons of continuous variables showing non-Gaussian distribution were performed using Mann-Whitney *U* test. We used GLM to compare MDS-UPDRS III and striatal SBRs between groups with age and sex as covariates for the comparison between PD and control groups, or with age, sex and disease duration for the comparison between PD subgroups. A LMM with random intercept was employed to compare annual SBR changes between groups. In LMM tests, we used baseline SBRs, disease duration at baseline, groups and interactions between baseline SBRs and groups as fixed effects, and patients as the random effect. Correction for multiple testing was performed using the Bonferroni method. Statistical significance was defined as *p* < 0.05.

### Reporting summary

Further information on research design is available in the Nature Research Reporting Summary linked to this article.

## Supplementary information

Reporting summary.

Supplementary information.

## Data Availability

Full database of the PPMI study is available at www.ppmi-info.org. The authors will provide de-identified and anonymized data of Gangnam Severance Hospital upon authorized request.

## Code availability

All analyses were performed using R software (version 3.6.1; r-project.org). Codes for data analysis in the present study are available upon request.
