# Author Correction: Genetic factors affecting dopaminergic deterioration during the premotor stage of Parkinson disease

**DOI:** 10.1038/s41531-022-00294-y

**Published:** 2022-03-09

**Authors:** Myung Jun Lee, Kyoungjune Pak, Han-Kyeol Kim, Kelly N. Nudelman, Jong Hun Kim, Yun Hak Kim, Junho Kang, Min Seok Baek, Chul Hyoung Lyoo

**Affiliations:** 1 Department of Neurology, Pusan National University Hospital, Pusan National University School of Medicine and Biomedical Research Institute, Busan, Republic of Korea; 2 Department of Nuclear Medicine, Pusan National University Hospital, Pusan National University School of Medicine and Biomedical Research Institute, Busan, Republic of Korea; 3 Department of Neurology, Gangnam Severance Hospital, Yonsei University College of Medicine, Seoul, Republic of Korea; 4 Department of Medical and Molecular Genetics, Indiana University School of Medicine, Indianapolis, IN USA; 5 Department of Neurology, National Health Insurance Service Ilsan Hospital, Goyang, Gyeonggi-do Republic of Korea; 6 Department of Anatomy, Pusan National University School of Medicine and Biomedical Research Institute, Yangsan, Gyeongsangnam-do Republic of Korea; 7 Department of Biomedical Informatics, Pusan National University School of Medicine, Yangsan, Gyeongsangnam-do Republic of Korea; 8 Interdisciplinary Program of Genomic Data Science, Pusan National University, Busan, Republic of Korea; 9 Department of Neurology, Wonju Severance Christian Hospital, Yonsei University Wonju College of Medicine, Wonju, Gangwon-do Republic of Korea

**Keywords:** Parkinson's disease, Neurodegeneration

Correction to: *npj Parkinson’s Disease* 10.1038/s41531-021-00250-2, published online 26 November 2021

In Table 1 of this article, the value in the column headed “GSH cohort: Controls (*n* = 71)” and in the row “CN, more affected” was incorrectly stated as “6.84 ± 1.24”, where the correct value is “5.84 ± 1.24”. In the column headed “GSH cohort: Controls (*n* = 71)” and in the row “PUT, more affected”, the value was incorrectly stated as “9.07 ± 1.27”, where the correct value is “8.07 ± 1.27”. The original article has been corrected.

