# Supplementary material for: Genetic factors affecting dopaminergic deterioration during the premotor stage of Parkinson disease
Source: NPJ Parkinsons Dis. 2021 Nov 26;7:104. doi: 10.1038/s41531-021-00250-2 (PMC8626486; doi:10.1038/s41531-021-00250-2)
Supplement: Supplementary file 1 — Reporting summary. [file 41531_2021_250_MOESM1_ESM.pdf]

nature portfolio

Corresponding author(s): Professor Chul Hyoung Lyoo

Last updated by author(s): Oct 20, 2021

Reporting Summary

Nature Portfolio wishes to improve the reproducibility of the work that we publish. This form provides structure for consistency and transparency in reporting. For further information on Nature Portfolio policies, see our Editorial Policies and the Editorial Policy Checklist.

Statistics

For all statistical analyses, confirm that the following items are present in the figure legend, table legend, main text, or Methods section.

n/a	Confirmed	
☐	☒	The exact sample size (n) for each experimental group/condition, given as a discrete number and unit of measurement
☐	☒	A statement on whether measurements were taken from distinct samples or whether the same sample was measured repeatedly
☐	☒	The statistical test(s) used AND whether they are one- or two-sided<br>*Only common tests should be described solely by name; describe more complex techniques in the Methods section.*
☐	☒	A description of all covariates tested
☐	☒	A description of any assumptions or corrections, such as tests of normality and adjustment for multiple comparisons
☐	☒	A full description of the statistical parameters including central tendency (e.g. means) or other basic estimates (e.g. regression coefficient) AND variation (e.g. standard deviation) or associated estimates of uncertainty (e.g. confidence intervals)
☐	☒	For null hypothesis testing, the test statistic (e.g. F, t, r) with confidence intervals, effect sizes, degrees of freedom and P value noted<br>*Give P values as exact values whenever suitable.*
☒	☐	For Bayesian analysis, information on the choice of priors and Markov chain Monte Carlo settings
☐	☒	For hierarchical and complex designs, identification of the appropriate level for tests and full reporting of outcomes
☐	☒	Estimates of effect sizes (e.g. Cohen's d, Pearson's r), indicating how they were calculated

Our web collection on statistics for biologists contains articles on many of the points above.

Software and code

Policy information about availability of computer code

Data collection	1. Open dataset of Parkinson's Progression Markers Initiative (PPMI) study (available at www.ppmi-info.org) 2. Authors own data: Gangnam severance Hospital
Data analysis	R software 3.6.1 (packages: ggplot2, dplyr, rms, Hmisc, gridExtra, patchwork, nlme, nlme4)

For manuscripts utilizing custom algorithms or software that are central to the research but not yet described in published literature, software must be made available to editors and reviewers. We strongly encourage code deposition in a community repository (e.g. GitHub). See the Nature Portfolio guidelines for submitting code & software for further information.

Data

Policy information about availability of data

All manuscripts must include a data availability statement. This statement should provide the following information, where applicable:
- Accession codes, unique identifiers, or web links for publicly available datasets
- A description of any restrictions on data availability
- For clinical datasets or third party data, please ensure that the statement adheres to our policy

We have stated in the manuscript that the dataset of Parkinson's Progression Markers Initiative is available at www.ppmi-info.org, and anonymized data of Gangnam Severance Hospital upon authorized request.

Field-specific reporting

Please select the one below that is the best fit for your research. If you are not sure, read the appropriate sections before making your selection.

☒ Life sciences ☐ Behavioural & social sciences ☐ Ecological, evolutionary & environmental sciences

For a reference copy of the document with all sections, see nature.com/documents/nr-reporting-summary-flat.pdf

Life sciences study design

All studies must disclose on these points even when the disclosure is negative.

Sample size	691 subjects (478 case, 213 controls)
Data exclusions	185 subjects
Replication	109 subjects (38 cases, 71 controls)
Randomization	N/A (the present study has observation design, and aims to compute a mathematical model.
Blinding	N/A (the present study has observation design, and aims to compute a mathematical model.

Reporting for specific materials, systems and methods

We require information from authors about some types of materials, experimental systems and methods used in many studies. Here, indicate whether each material, system or method listed is relevant to your study. If you are not sure if a list item applies to your research, read the appropriate section before selecting a response.

Materials & experimental systems

n/a	Involved in the study	
☒	☐	Antibodies
☒	☐	Eukaryotic cell lines
☒	☐	Palaeontology and archaeology
☒	☐	Animals and other organisms
☐	☒	Human research participants
☐	☒	Clinical data
☒	☐	Dual use research of concern

Methods

n/a	Involved in the study	
☒	☐	ChIP-seq
☒	☐	Flow cytometry
☒	☐	MRI-based neuroimaging

Human research participants

Policy information about studies involving human research participants

Population characteristics	Patients with Parkinson disease, older than 30 years of age, diagnosed with Parkinson's disease within the last 2 years, having a Hoehn&Yahr stage not greater than II, being untreated, showing striatal dopaminergic dysfunction on dopamine transporter stage. (detailed inclusion criteria are shown at ppmi-info.org.)
Recruitment	Subjects were recruited at 33 clinical sites in 11 countries. (2010 - 2018)
Ethics oversight	The Parkinson's Progression Markers Initiative (PPMI) study is registered at ClinicalTrials.gov (NCT01141023). Each PPMI site received approval from an ethics committee on human experimentation prior to study initiation. The Institutional Review Board of Gangnam Severance Hospital also approved the present study for cross-validation. Written informed consent for the research was obtained from all individuals participating in the PPMI study and GSH cohort.

Note that full information on the approval of the study protocol must also be provided in the manuscript.

Clinical data

Policy information about clinical studies

All manuscripts should comply with the ICMJE guidelines for publication of clinical research and a completed CONSORT checklist must be included with all submissions.

Clinical trial registration	NCT01141023
Study protocol	The PPMI study is a longitudinal observational study, and aims to find a biomarker for the progression of Parkinson's disease.

Data collection	biofluid markers, neuroimaging (dopamine transporter scan, brain MRI and others), clinical rating scales
Outcomes	N/A (the study is currently ongoing)
