# Supplementary material for: Genetic factors affecting dopaminergic deterioration during the premotor stage of Parkinson disease
Source: NPJ Parkinsons Dis. 2021 Nov 26;7:104. doi: 10.1038/s41531-021-00250-2 (PMC8626486; doi:10.1038/s41531-021-00250-2)
Supplement: Supplementary file 2 — Supplementary information. [file 41531_2021_250_MOESM2_ESM.pdf]

Supplementary Figure 1.

RCS fit and estimated trajectory using DAT SBRs of posterior putamen in GSH sPD patients.

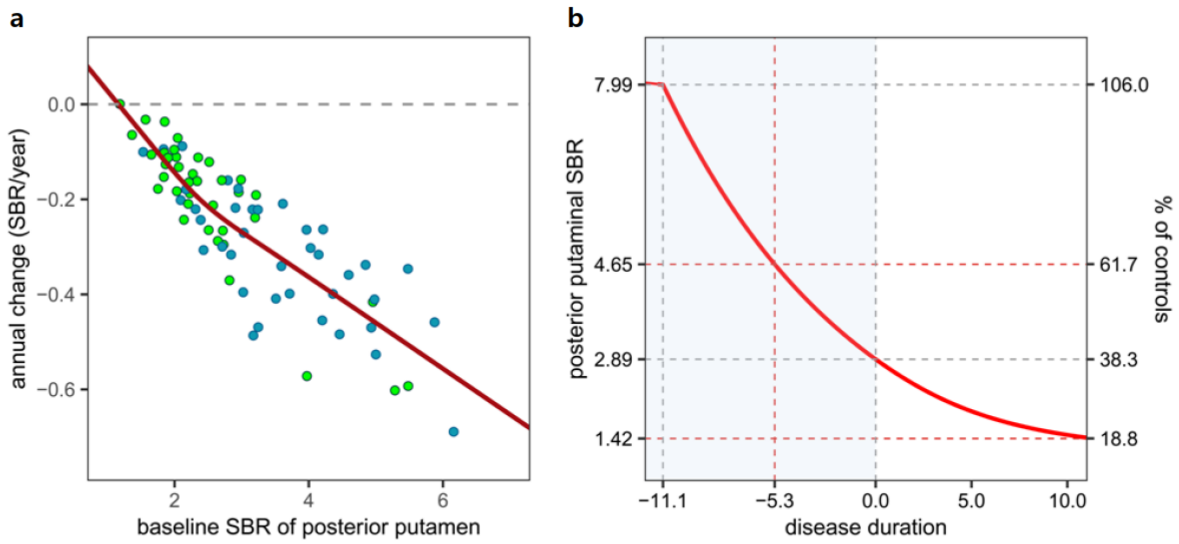

Figure legend. RCS curve fit between baseline SBR of posterior putamen and annual change rates (A) and estimated temporal trajectory of DAT SBR (B) in GSH cohort. DAT decline began 11.1 years prior to motor onset. Estimated DAT availability reached 60% of age-adjusted controls 5.3 years before the onset of motor features. 11.4 years later motor onset, estimated level decreased as 20% of age-adjusted controls.

Supplementary Figure 2.

Comparison of DAT trajectories between GRS quantiles

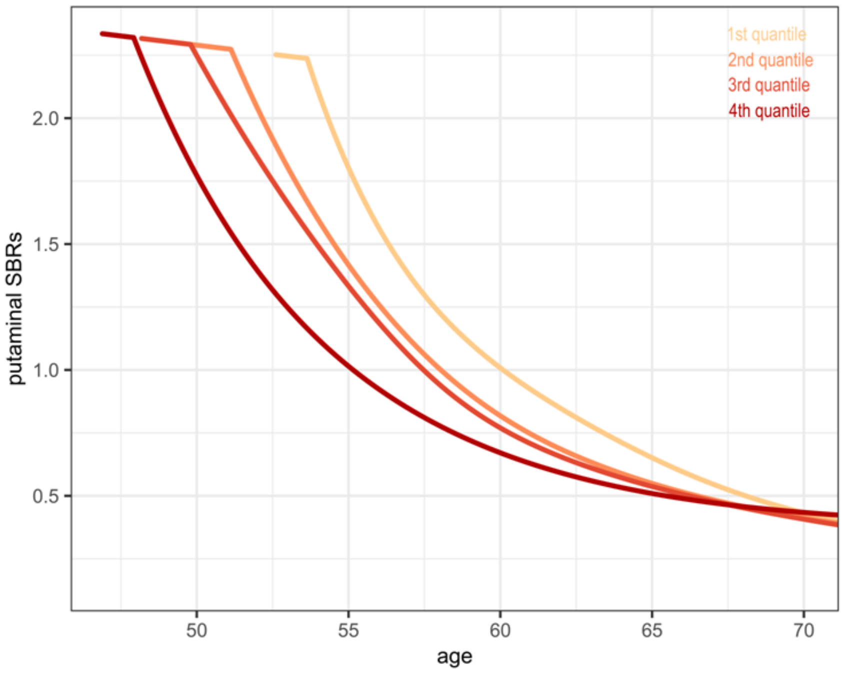

	Quantile 1	Quantile 2	Quantile 3	Quantile 4
Duration of premotor stage (years)	9.4	9.8	10.3	11.8
Age at motor onset	61.1	60.4	60.0	58.0
Age at onset of DAT decline	51.7	50.6	49.7	46.2
SBR reduction rate during premotor stage (SBR/year)	0.16	0.16	0.15	0.14
SBR at onset of DAT decline	2.24	2.27	2.29	2.32
SBR at motor onset	0.78	0.75	0.76	0.68
Number of PD patients	83	83	83	83

Figure legend. Estimated DAT trajectories of GRS quantiles in sPD patients. Higher GRS quantile was associated with longer premotor stage and a trend of slower progression of dopaminergic dysfunction.

Supplementary Figure 3.

Ethnic classification of PPMI subjects.

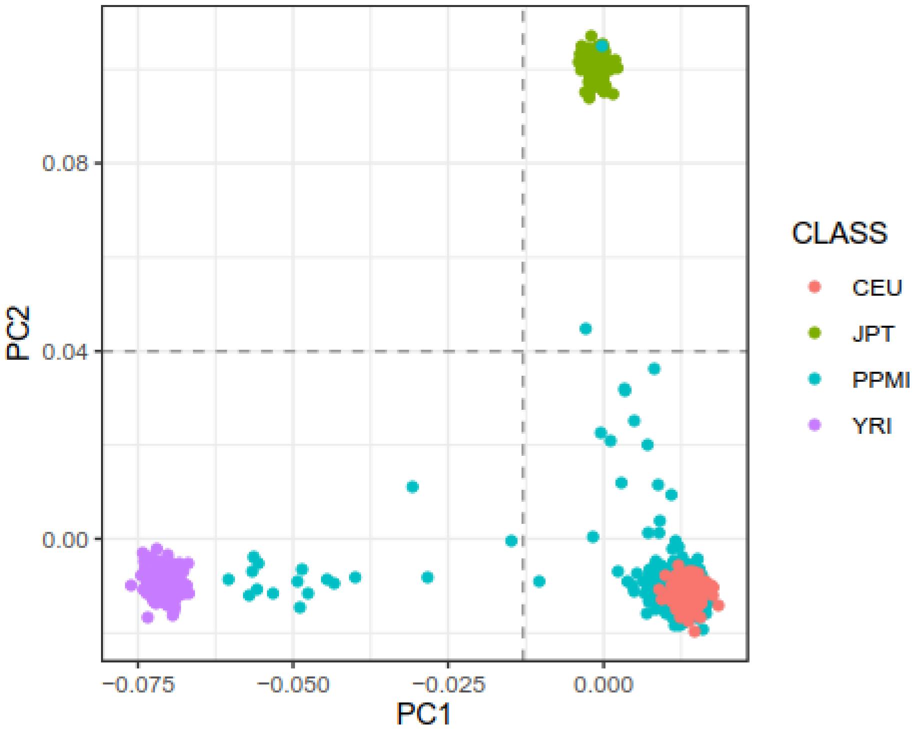

Figure legend. Scatter plot showing principal components (PCs) obtained from NEUROX chip data of PPMI and HAPMAP genetic data. CEU: 30 trios of northern and western European ancestry living in Utah from the Centre d'Etude du Polymorphisme Humain collection; JPT: 45 unrelated Japanese individuals in Tokyo, Japan; YRI: 30 mother-father-adult child trios from the Yoruba in Ibadan, Neigeria; PPMI: Parkinson's Progression Markers Initiative. Dashed line represents cut off value for European ancestry.

Supplementary Figure 4.

Schematic explanation of the Euler method and its modification

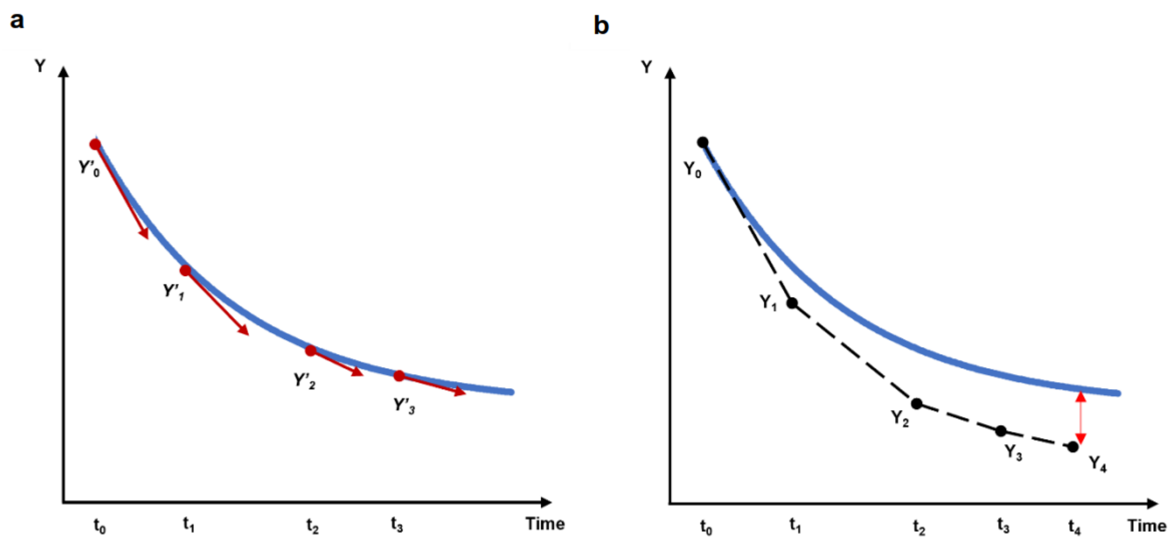

Figure legends. a) When function Y with unknown temporal pattern is given (blue line), the Euler method predicts a next coming value (Y_{n+1}) using the slope (first derivative for time interval ΔT; Y'_n) at previous value Y_n ($Y_{n+1} = Y_n + Y'_n \times \Delta T$). **b)** In summary, the Euler method approximates total trajectory using consecutive tangent lines (dashed lines). Error between true values of function Y (blue line) and estimated value (dots) becomes greater when the time interval (ΔT) increases, thus modified Euler method uses the mean value of the first derivatives (*Y'*) within interval. ($Y_{n+1} = Y_n + [(Y'_n + Y'_{n+1}]/2] \times \Delta T$)

Supplementary Figure 5.

Steps for obtaining an outcome of differential equation.

a

BL	Linear fit
B_1	R_1 x disease duration + $Intercept_1$
B_2	R_2 x disease duration + $Intercept_2$
B_3	R_3 x disease duration + $Intercept_3$
B_4	R_4 x disease duration + $Intercept_4$
·	.
·	.

BL = baseline; R = slope of liner model

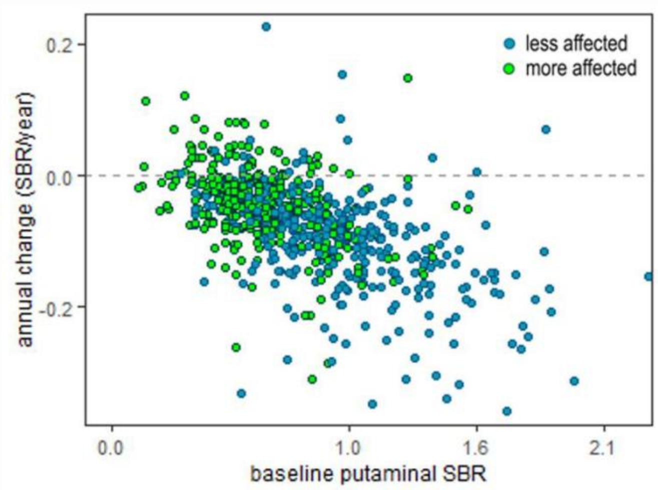

b

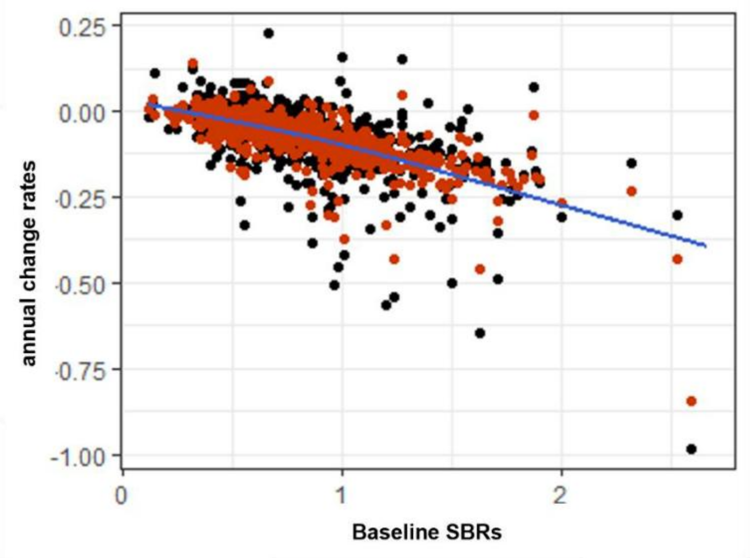

Red dot = Predicted value from LMM
Black dot = Actual SBRs and slopes
Blue line = restricted cubic spline fit for predicted values from LMM model

Figure legend. a) We calculated the annual change rates of each putaminal SBR, and plotted annual changes in SBRs against baseline values. Plotted values are represent baseline SBRs and change rates in sporadic PD group in the present study. **b)** Mixed effect model with cubic splines (knot location: 5-, 35-, 65- and 95-percentile of ebaseline SBRs) was applied, and predicted values were plotted. Then the curve showing association between baseline SBRs and predicted change rates was acquired by applying restricted cubic spline function.

Supplementary Figure 6.

Steps for obtaining trajectory of DAT SBRs over time.

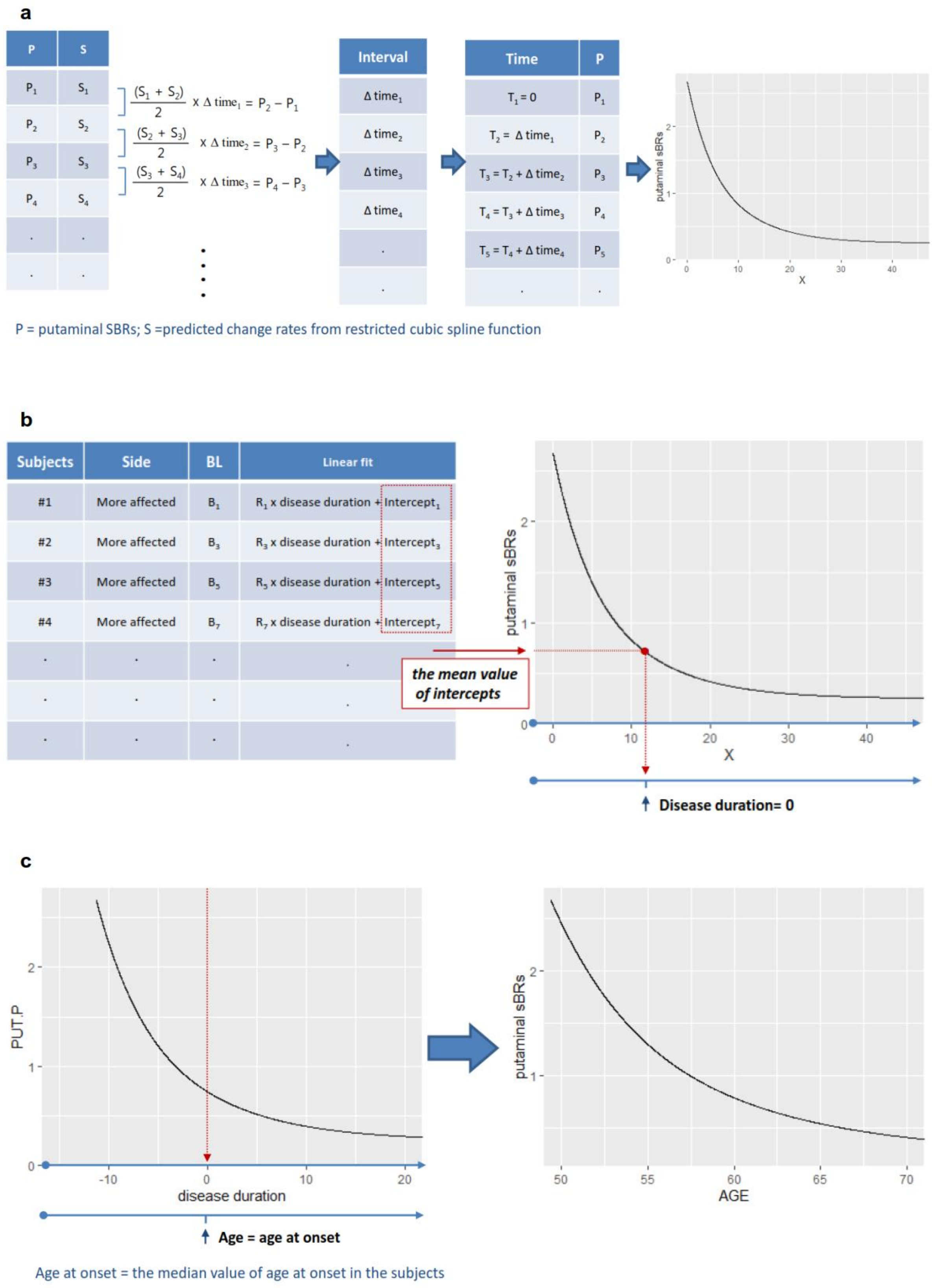

Figure legend. a) Estimating the shape of DAT SBRs over time. The modified Euler method

calculates a next-coming value and the shape of an unknown curve with given a differential equation when a unit time elapses. In the present study, the result of differential equation was obtained from restricted cubic spline fit (Supplementary Figure 3), so we can calculate a time elapsed as DAT SBRs decrease. **b)** Anchoring the trajectory along with disease duration. We applied disease duration = 0 (at the motor onset) for linear regression models of individual putamen at more affected side. The mean estimated putaminal SBR at the onset of motor symptoms was calculated to anchor the trajectory on the time point for the onset. **c)** Anchoring the trajectory along with age. To convert x-axis to age, "age" was calculated by adding the median value of age at onset (AAO) to disease duration (age = disease duration + the median AAO of each PD group).

Supplementary Figure 7.

Combination of non-PD and PD state models

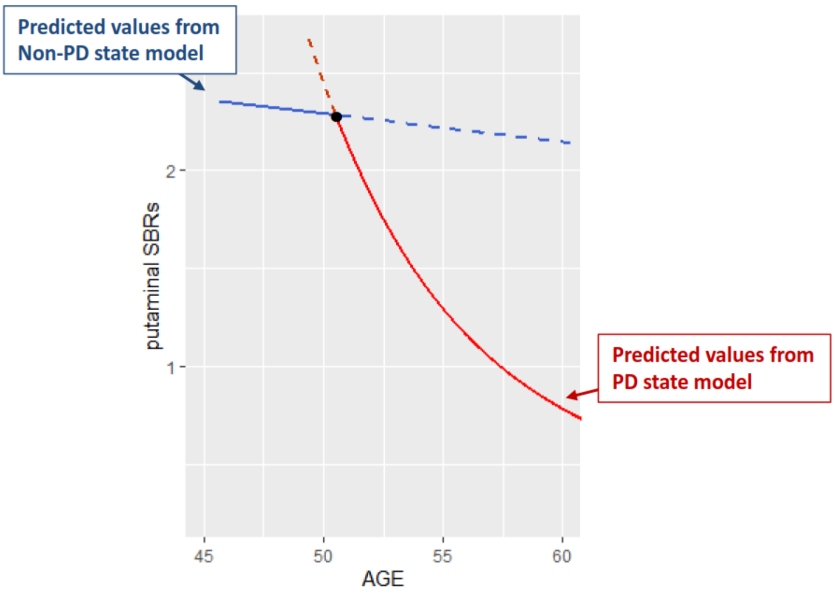

Figure legend. At the final step of estimating the DAT trajectory, we calculated the intersection point between non-PD and PD state models. The intersection point was defined as onset of striatal dopaminergic dysfunction.

Supplementary Table 1.

Effect of genetic factors and age at onset (AAO) on the annual change rates of putaminal DAT SBRs

**Main effect**	**Reference group**	**Estimate**	**SE**	**t**	***p***
**AAO**		-0.0012	0.0007	-1.7176	0.087
**GRS (low GRS)**	high GRS	-0.0148	0.0149	-0.9944	0.321
***GBA* PD *vs.* sPD (sPD)**	*GBA* PD	-0.0637	0.0205	-3.1108	0.006[a*]
***GBA* PD *vs.* *LRRK2* PD (LRRK2 PD)**	*GBA* PD	-0.0426	0.0261	-1.6367	0.315 [a]
**sPD vs. *LRRK2* PD (sPD)**	*LRRK2* PD	-0.0279	0.0187	-1.4968	0.405 [a]
**Interaction with baseline SBRs**					
**AAO**		0.0015	0.0007	2.1378	0.033[*]
**GRS (low GRS)**	high GRS	0.0095	0.0157	0.6053	0.545
***GBA* PD *vs.* sPD (sPD)**	*GBA* PD	0.0917	0.0217	4.2272	< 0.001 [a *]
***GBA* PD *vs.* *LRRK2* PD (*LRRK2* PD)**	*GBA* PD	0.0652	0.0297	2.1950	0.090 [a]
**sPD vs. *LRRK2* PD (sPD)**	*LRRK2* PD	0.0294	0.0217	1.3540	0.528 [a]

Results of linear mixed effect models covariated with AAO and disease duration; t= t-statistics in linear mixed effect model; GRS = genetic risk score; AAO = treated as continuous variable;a = corrected for multiple testing (Bonferroni correction);* = p < 0.05.

Supplementary Table 2.

Comparison of clinical and imaging characteristics between excluded and included PPMI PD subjects

	**Excluded subset**	**Included subset**	*p*
Age[a]	64.0 ± 10.3	61.7 ± 9.6	0.014*
Sex ratio (M:F) [b]	90:68	306:195	0.357
Disease duration[c]	4.1 ± 5.4	2.5 ± 2.5	< 0.001*
MDS-UPDRS III, sum[d]	22.2 ± 10.8	20.7 ± 9.3	0.506
H&Y stage [b]	59/80/8	208/263/8	0.060
APOE e4 risk allele [b]	19/51 (37.2%)	91/350 (26.0%)	0.092
Striatal DAT SBRs[d]			
Caudate nucleus, less affected side	2.007 ± 0.722	2.103 ± 0.611	0.355
Caudate nucleus, more affected side	1.802 ± 0.662	1.826 ± 0.556	0.904
Putamen, less affected side	0.965 ± 0.481	0.968 ± 0.388	0.610
Putamen, more affected side	0.719 ± 0.425	0.671 ± 0.288	0.059

Mean ± SD; DAT = dopamine transporter; SBR = specific binding ratio; a= independent t-test; b = chi-square test; c = Mann-Whitney U test; d = linear regression covariated age, sex and disease duration; * = *p*-value < 0.05

Supplementary Table 3. List of SNPs for the calculation of genetic risk scores (GRSs)

SNP	Location (hg19)	Nearest gene	Alleles*	MAF	Meta-OR
rs10797576	chr1:232664611	SIPA1L2	T>C (T)	0.092	1.13
rs10906923	chr10:15569598	ITGA8	C>A (C)	0.300	0.93
rs11060180	chr12:123303586	CCDC62	G>A (G)	0.483	0.91
rs11158026	chr14:55348869	GCH1	T>C (T)	0.367	0.91
rs11343	chr16:19279464	SYT17	T>G (T)	0.475	1.08
rs114138760	chr1:154898185	PMVK	C>G (C)	0.017	1.50
rs115185635	chr3:87520857	intergenic	C>G (C)	0.050	1.79
rs11724635	chr4:15737101	BST1	C>A (C)	0.433	0.89
rs117896735	chr10:121536327	INPP5F	A>G (A)	0.017	1.77
rs118117788	chr10:121710488	MIR4682 [-7537bp]	T>C (T)	0.025	1.58
rs11868035	chr17:17715101	SREBF1	A>G (A)	0.258	0.94
rs12456492	chr18:40673380	RIT2	G>A (G)	0.375	1.10
rs12497850	chr3:48748989	IP6K2	G>T (G)	0.392	0.93
rs12637471	chr3:182762437	MCCC1	A>G (A)	0.242	0.84
rs1293298	chr8:11712443	CTSB	C>A (C)	0.233	0.92
rs13294100	chr9:17579690	SH3GL2	T>G (T)	0.350	0.91
rs14235	chr16:31121793	BCKDK	A>G (A)	0.450	1.10
rs17649553	chr17:43994648	MAPT	T>C (T)	0.217	0.77
rs1955337	chr2:169129145	STK39 [+24494bp]	T>G (T)	0.083	1.21
rs199347	chr7:23293746	GPNMB	G>A (G)	0.392	0.90
rs2280104	chr8:22525980	BIN3	T>C (T)	0.358	1.08
rs2414739	chr15:61994134	intergenic	G>A (G)	0.283	0.90
rs2694528	chr5:60273923	NDUFAF2	C>A (C)	0.117	1.14
rs329648	chr11:133765367	MIR4697 [-3032bp]	T>C (T)	0.333	1.11
rs34043159	chr2:102413116	MAP4K4	C>T (C)	0.383	1.08
rs34311866	chr4:951947	TMEM175	C>T (C)	0.150	1.26
rs34884217	chr4:944210	TMEM175	C>A (C)	0.100	0.74
rs353116	chr2:166133632	SCN2A	T>C (T)	0.358	0.93
rs356181	chr4:90626139	SNCA [-19111bp]	A>G (A)	0.467	0.78
rs356182	chr4:90626111	SNCA [-19139bp]	G>A (G)	0.417	1.34
rs3793947	chr11:83544472	DLG2	A>G (A)	0.375	0.91
rs3910105	chr4:90682571	SNCA	G>A (G)	0.458	0.89
rs4073221	chr3:18277488	TBC1D5	G>T (G)	0.133	1.11
rs4653767	chr1:226916078	ITPKB	C>T (C)	0.283	0.92
rs4784227	chr16:52599188	TOX3 [+17474bp]	T>C (T)	0.208	1.09
rs55785911	chr20:3153503	UBOX5 [+12661bp]	A>G (A)	0.417	0.91
rs591323	chr8:16697091	intergenic	A>G (A)	0.325	0.91
rs6430538	chr2:135539967	intergenic	T>C (T)	0.408	0.88
rs6812193	chr4:77198986	FAM47E	T>C (T)	0.392	0.91
rs71628662	chr1:155359992	ASH1L	T>C (T)	0.018	0.52
rs76904798	chr12:40614434	LRRK2	T>C (T)	0.158	1.16
rs78738012	chr4:114360372	CAMK2D [-11816bp]	C>T (C)	0.150	1.14
rs8005172	chr14:88472612	GPR65	T>C (T)	0.400	1.07
rs823118	chr1:205723572	NUCKS1 [+4168bp]	C>T (C)	0.450	0.89
rs9468199	chr6:27681215	intergenic	A>G (A)	0.108	1.10

Alleles = effect allele > alternative allele (minor allele), MAF = minor allele frequencies in 1000G

database (Caucasian), meta-OR = meta-odds ratio of PD in the public database (pdgene.org)
